# Characterization of CA-MRSA TCH1516 exposed to nafcillin in bacteriological and physiological media

**DOI:** 10.1038/s41597-019-0051-4

**Published:** 2019-04-26

**Authors:** Saugat Poudel, Hannah Tsunemoto, Michael Meehan, Richard Szubin, Connor A. Olson, Anne Lamsa, Yara Seif, Nicholas Dillon, Alison Vrbanac, Joseph Sugie, Samira Dahesh, Jonathan M. Monk, Pieter C. Dorrestein, Joseph Pogliano, Rob Knight, Victor Nizet, Bernhard O. Palsson, Adam M. Feist

**Affiliations:** 1 Department of Bioengineering, University of California, San Diego, La Jolla, USA; 2 Division of Biological Sciences, University of California San Diego, La Jolla, CA 92093 USA; 3 Collaborative Mass Spectrometry Innovation Center, University of California, San Diego, La Jolla, California USA; 4 Skaggs School of Pharmacy and Pharmaceutical Sciences, University of California San Diego, La Jolla, CA USA; 5 Department of Pediatrics, University of California, San Diego, La Jolla, CA USA; 6 Novo Nordisk Foundation Center for Biosustainability, Technical University of Denmark, Kemitorvet, Building 220, 2800 Kongens Lyngby, Denmark; 7 Center for Marine Biotechnology and Biomedicine, Scripps Institution of Oceanography, University of California San Diego, La Jolla, CA 92093 USA; 8 Department of Computer Science and Engineering, University of California San Diego, La Jolla, CA 92093 USA; 9 Center for Microbiome Innovation, University of California San Diego, La Jolla, CA 92093 USA

**Keywords:** Pathogens, Antibiotics, RNA sequencing, Metabolomics

## Abstract

Cation adjusted-Mueller Hinton Broth (CA-MHB) is the standard bacteriological medium utilized in the clinic for the determination of antibiotic susceptibility. However, a growing number of literature has demonstrated that media conditions can cause a substantial difference in the efficacy of antibiotics and antimicrobials. Recent studies have also shown that minimum inhibitory concentration (MIC) tests performed in standard cell culture media (e.g. RPMI and DMEM) are more indicative of *in vivo* antibiotic efficacy, presumably because they are a better proxy for the human host’s physiological conditions. The basis for the bacterial media dependent susceptibility to antibiotics remains undefined. To address this question, we characterized the physiological response of methicillin-resistant *Staphylococcus aureus* (MRSA) during exposure to sub-inhibitory concentrations of the beta-lactam antibiotic nafcillin in either CA-MHB or RPMI + 10% LB (R10LB). Here, we present high quality transcriptomic, exo-metabolomic and morphological data paired with growth and susceptibility results for MRSA cultured in either standard bacteriologic or more physiologic relevant medium.

## Abstract (summary)

**Table:** 

Design Type(s)	replicate design • transcription profiling design • sequence analysis objective
Measurement Type(s)	transcription profiling assay • cellular morphology • exo-metabolome • growth
Technology Type(s)	RNA sequencing • fluorescence microscopy • liquid chromatography-tandem mass spectrometry • high performance liquid chromatography • Optical Density Measurement
Factor Type(s)	culture medium • biological replicate • experimental condition
Sample Characteristic(s)	Staphylococcus aureus • culturing environment

Machine-accessible metadata file describing the reported data (ISA-Tab format)

## Background & Summary

The emergence of antimicrobial resistance amongst bacterial pathogens is quickly exhausting our current repertoire of efficacious treatment options. With growing biological and economic limitations on traditional approaches to antibiotic therapy, infectious diseases are on pace to reclaim their status as the leading cause of human mortality worldwide^1^. With the acquisition of multidrug resistance and its rapid spread outside of the nosocomial environment, community-associated methicillin-resistant S. *aureus* (CA-MRSA) strains have exacerbated the burden imposed on modern healthcare^2^. While there have been extensive efforts to develop novel therapeutics we still do not fully understand the bacterial physiological transformations that occur to promote the evolution of antibiotic resistance.

CA-MHB is the standard media used for determining the minimum inhibitory concentration (MIC) of antibiotics against *S. aureus* in both clinical and research settings^3^. It is designed for non-selective and rapid growth of microbes in ideal conditions. However, many recent studies have shown that a pathogen’s response to antimicrobial compounds is dependent on the media conditions in which they were tested and the consequent cellular state^4–6^. Furthermore, Ersoy *et al.* demonstrated that the accuracy of antibiotic susceptibility testing can be improved by using cell culture media that are designed to mimic host physiological conditions, calling into question the prevalent usage of CA-MHB^7^. Though many cellular processes have been linked to changes in MIC, it is still unclear which (if any) of the proposed mechanisms play a role in the differences we observe between bacteriological and physiological media^4,8^.

**Fig. 1:**
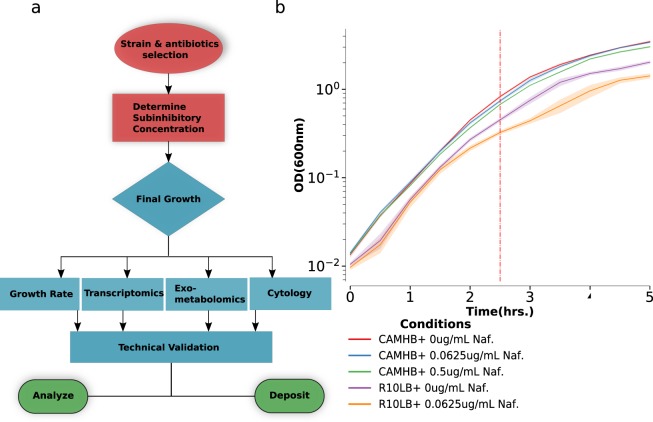
Data collection. (**a**) Schematic depicting the workflow used to generate the multi-omic data. (**b**) Plot of growth curves during sample collection.Cells were grown for five hours in each condition. At 2.5 hour mark (red dashed line), samples were collected for BCP and Rna seq while samples for exo-metabolomics and growth rate were collected every 30 minutes. The shaded region represents 95% confidence intervals (CI) of the OD600 measurements from three biological replicates.

To address this question, we characterized the response of CA-MRSA isolate TCH1516 to different sub-inhibitory concentrations of nafcillin in CAMHB and RPMI + 10% LB (R10LB) using a workflow designed for multi-omic data collection (Fig. 1a). In accordance with previous findings, we found that the MIC of nafcillin dropped from 12.8 ug/mL in CA-MHB to 0.2 ug/mL in R10LB^7^. Next, we grew the cells in several sub-inhibitory concentrations of nafcillin and chose the conditions that allowed for lowered but continued steady state growth for further analysis. The growth rates under these conditions were checked repeatedly to ensure that they were highly reproducible (Fig. 1b)^9^. This allowed us to streamline our protocol by letting us take samples at the same time point across the two media conditions. Sampling at a consistent time point also had an added advantage of having equal exposure time to nafcillin, which has a time-dependent bactericidal effect^9^. Under these conditions, we characterized TCH1516 response with growth rate measurements, RNA sequencing, exo-metabolomics (HPLC and LC-MS) and Bacterial Cytological Profiling (BCP). This dataset provides multi-omic coverage of cellular response to antibiotic in conditions that lead to drastically different efficacy of the drug. When used in the framework of emerging genome scale models and integration methods, it will provide a more complete understanding of emerging beta-lactam resistance.

## Methods

### Culture and Growth Conditions

Standard bacteriological media MHB (Sigma-Aldrich) was supplemented with 25 mg/L Ca^2+^ and 12.5 mg/L Mg2+ (CA-MHB). Eukaryotic cell culture media Roswell Park Memorial Institute 1640 (RPMI) (Thermo Fisher Scientific) was supplemented with 10% LB (R10LB). Broth microdilution was performed to determine the nafcillin MIC in each media condition. On the day of the experiment, overnight cultures of CA-MRSA TCH1516 were diluted to a starting OD600 of 0.01 into fresh media and grown at 37 °C with stirring to OD600 0.4. This preculture was then diluted back to OD600 0.01 into fresh media containing no drug or sub-inhibitory concentrations of nafcillin relative for each media type. Growth was monitored by obtaining OD600 readings every 30 min for 5 hr. Three biological replicates were collected for the study, each derived from different colony and overnight culture.

### cDNA library preparation and RNA sequencing

After 2.5 hours of growth, 3 mL samples were taken for RNA sequencing and added to tubes containing 6 mL RNAprotect. After incubation, they were centrifuged to remove the supernatant. RNA was extracted from the pelleted cells using a ‘Quick RNA Fungal/Bacterial Microprep’ kit developed by Zymo Research. The cells were mechanically lysed with a Roche MagNa Lyser instrument and DNA was removed with DNase I during the RNA purification. RNA quality was checked with an Agilent Bioanalyzer instrument and ribosomal RNA was removed using an Illumina Ribo-Zero kit. The remaining RNA was used to build a cDNA library for sequencing using a KAPA Stranded RNA-seq Library Preparation Kit. The kit was used for RNA fragmentation, sequencing adapter ligation, and library amplification. The generated cDNA libraries were sent for Illumina sequencing on a HiSeq 4000.

### RNA sequencing analysis

The phred quality scores for illumina sequencing were generated using Fastqc package^10^. Bowtie2 was used to align the raw reads to TCH1516 genome (NC_010079.1) and to calculate alignment percentage^11,12^. The aligned reads were then normalized to transcripts per million (TPM) with DESeq2. The ComBat module within the sva package of R was used to correct for batch effects^13,14^. Distance matrix for hierarchical clustering were calculated with sklearn package^15^.

### Bacterial Cytological Profiling

At the 2.5 hour mark, samples were taken for fluorescence microscopy, similar to previously described with modifications^16^. In brief, 8 µL cells were added to 2 µL dye mix containing 10 µg/mL DAPI, 2.5 µM SYTOX Green, and either 20 µg/mL WGA-555 or WGA-Rhodamine in 1x T-base. The sample was then transferred to a glass slide containing an agarose pad (20% media, 1.2% agarose) and imaged on an Applied Precision DV Elite epifluorescence microscope with a CMOS camera. The exposure times for each wavelength were as follows, TRITC/Cy-5 = 0.025 s, FITC/FITC = 0.01 s, DAPI/DAPI = 0.015 s, and were kept constant for all images.

**Fig. 2:**
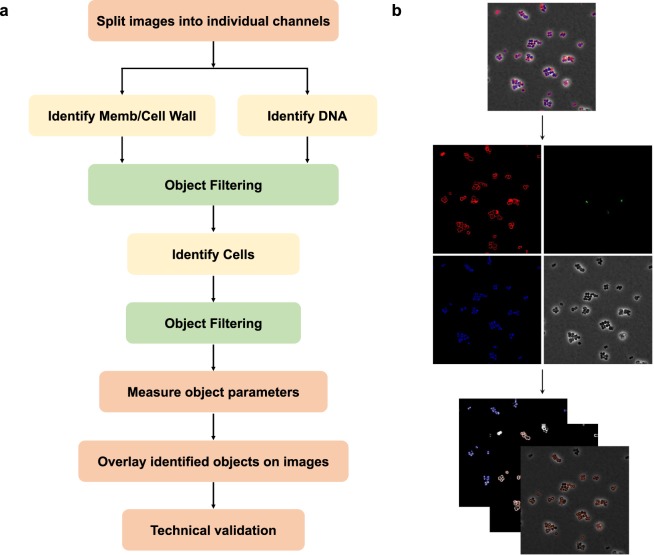
Image analysis pipeline for cell morphology. (**a**) Schematic of cytology data pipeline using CellProfiler 3.0. (**b**) Representation of image analysis pipeline. Undeconvolved and deconvolved composite images are split into individual channels. Images were thresholded and filtered WGA and DAPI channels to generate segmentation masks for identified objects.

Deconvolved images were adjusted using FIJI (ImageJ 1.51w) and Adobe Photoshop (2015.1) to remove background in WGA and DAPI channels and to ensure that cell and DNA objects are within the highest intensity quartile. These images were then processed using a custom CellProfiler 3.0 pipeline that individually thresholded and filtered WGA and DAPI channels to obtain segmentation masks for the cell wall, DNA and entire cell. Objects identified in this manner were further processed in CellProfiler to obtain a total of 5285 features^17–19^. Prior to analysis, feature selection is necessary to create a subset of relevant features as to minimize redundancy within the dataset. The summary of processing steps and an example image are presented in Fig. 2.

### Untargeted Liquid Chromatography Mass Spectrometry Data Acquisition

Following dilution of the preculture of CA-MRSA TCH1516 into fresh media, approximately 400 µL of liquid media containing cells were collected at 30 minutes intervals (at the same time as samples for OD600 measurements) from each of the samples. Growth media was syringe-filtered through 0.22 µm disc filters (Millex-GV, MilliporeSigma) to remove cells. The filtered growth media was collected and stored at −80 °C until analysis by liquid chromatography mass spectrometry (LC/MS). For LC/MS analysis, samples were subjected to chromatographic separation using an UltiMate 3000 UHPLC system (Thermo Scientific). Chromatographic separations were achieved using a 50 mm × 2.1 mm Kinetex 2.6 micron polar-C18 column (Phenomenex) held at a fixed temperature of 30 °C within an actively heated column compartment. Samples were injected onto the LC column via thermostatted autosampler maintained at 4 °C. For samples containing RPMI + 10% LB media the injection volume was 5 µl, while the injection volume was 2 µl for samples containing CA-MHB to prevent excessive oversaturation of the mass spectrometer detector due to the higher concentrations of many molecules in the CA-MHB media.

After injection, the sample components were eluted from the LC column into the mass spectrometer using a flow rate of 0.5 mL/min and the following mobile phases: Mobile phase A was LC/MS grade water with 0.1% formic acid (v/v) and mobile phase B was LC/MS grade acetonitrile with 0.1% formic acid (v/v). The LC gradient program was as follows: 0–1.0 min 5%B, 1.0–5.0 min 5–35%B, 5.0–5.5 min 35–100%B, 5.5–6.0 min 100%B, and 6.0–6.5 min 100–5%B followed by 5 minutes of re-equilibration at 5%B. Mass spectrometric data was acquired using a Bruker Daltonics maXis Impact quadrupole-time-of-flight (qTOF) mass spectrometer equipped with an Apollo II electrospray ionization (ESI) source and controlled via otofControl v4.0.15 and Hystar v3.2 software packages (Bruker Daltonics). The mass accuracy of the maXis instrument was first externally calibrated using a calibration solution of sodium formate which provided >21 reference m/z’s between 50–1500 m/z of the mass spectrum in both positive and negative polarities (reference m/z list provided within instrument control software). Sodium formate solution was prepared using 9.9 ml of 50/50% isopropanol/water, plus 0.2% formic acid, and 100 μl of 1 M NaOH. During infusion of all samples, the mass accuracy of the instrument was maintained to <10 ppm via constant introduction of and internal calibrant, or “lock mass”, in the form of hexakis (1H,1H,2H-difluoroethoxy)phosphazene (SynQuest Labs, Inc.). During positive polarity runs the lock mass compound was detected as the ion m/z 622.028960 (C_12_H_19_F_12_N_3_O_6_P_3_^+^) and in negative polarity the lock mass compound formed the ion m/z 556.001951 (C_10_H_15_F_10_N_3_O_6_P_3_^−^).

Instrument source parameters were set as follows: nebulizer gas (Nitrogen) pressure, 2 Bar; Capillary voltage, 3,500 V; ion source temperature, 200 °C; dry gas flow, 9 L/min. The global mass spectral acquisition rate was set at 3 Hz. The instrument transfer optics were tuned as follows: Ion funnel 1 & 2 RFs of 250 Vpp (volts peak-to-peak), hexapole RF of 100 Vpp, quadrupole ion energy of 5 eV, collision quadrupole energy of 5 eV, and a TOF pre-pulse storage of 7.0 µsecs. The post-collision quadrupole RF and TOF transfer time were stepped across four values per MS scan. The collision RF was stepped at 450, 550, 800, 1100 Vpp. The transfer time was stepped at 70, 75, 90, 95 µsecs. All samples were run twice, once under positive polarity settings and once under negative polarity settings.

Following acquisition of the LC/MS data, lock mass calibration was applied to all data files in order to apply a linear correction calibration to all m/z values recorded in each mass spectrum. The application of this mass correction was applied automatically via the Bruker Daltonics Compass Data Analysis software (ver. 4.3.110), using the m/z of the hexakis (1H,1H,2H-difluoroethoxy) phosphazene as the reference lock mass calibration compound. Following lock mass re-calibration of the data, all files were converted from the Bruker Daltonics proprietary format (.d) and exported to an open data format known as .mzXML. All data herein was deposited to MassIVE^20^.

### Targeted High-Performance Liquid Chromatography

For organic acid and carbohydrate detection, samples were collected every 30 minutes and filtered as described above. The filtered samples were loaded onto a 1260 Infinity series (Agilent Technologies) high-performance liquid chromatography (HPLC) system with an Aminex HPX-87H column (Bio-Rad Laboratories) and a refractive index detector. The system was operated using ChemStation software. The HPLC was run with a single mobile phase composed of HPLC grade water buffered with 5 mM sulfuric acid (H_2_SO4). The flow rate was held at 0.5 mL/minute, the sample injection volume was 10 uL, and the column temperature was maintained at 45 °C. The identities of compounds were determined by retention time comparison to standard curves of acetate, ethanol, glucose, lactate, pyruvate, and succinate. The peak area integration and resulting chromatograms were generated within ChemStation and compared to that of the standard curves in order to determine the concentration of each compound in the samples. These final concentration values were deposited to MassIVE database^20^.

### Exclusion Criteria

Some samples with low concentrations of antibiotics were excluded due to lack of statistically significant change in measured phenotypes. These measurements are available in public databases under same project numbers as those included in this manuscript or upon request.

## Data Records

The growth-rate data has been deposited in figshare^9^. HPLC, mass spectrometry as well as the cytological profiling data can be found in MassIVE^20^. The pipeline used to process and quality check RNA sequencing data has been deposited in figshare^21,22^. Raw FASTQ sequencing files for RNA sequencing have been deposited to the NCBI Sequence Read Archive^23^. The processed RNA sequencing file containing raw and ComBat corrected TPM and counts were deposited in Gene Expression Omnibus^24^. The summary statistics from RNA sequencing pipeline was compiled and submitted to figshare^25^.

## Technical Validation

### RNA sequencing

Each library was run on an Agilent Technologies TapeStation to check for correct size distribution and for the removal of unincorporated primers and adapters. After pooling libraries with distinct barcodes, a final size selection was performed with SPRI beads to remove sub-detectable PCR primer carryover. This final pool was checked again on the TapeStation prior to being run on the Illumina Hiseq 4000.

**Fig. 3:**
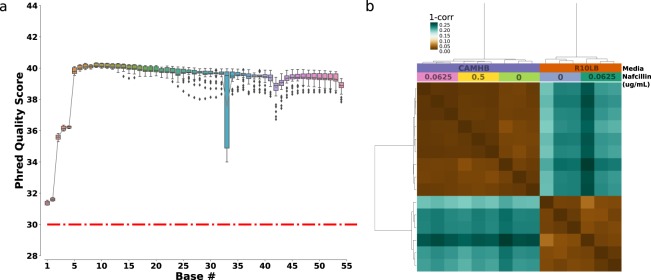
RNA sequencing technical validation. (**a**) A distribution plot of base wise phred quality score of all samples in the dataset. The red dashed line at the score of 30 corresponds to 99.9% accuracy in base calling. (**b**) Clustering of samples based on Spearman correlation of sample TPMs.

The quality of sequenced reads, as measured by average Phred Scores, was greater than 30 across all samples (Fig. 3a). These scores correspond to base calling accuracy of at least 99.9%, though for most bases the scores were much higher. These raw fastq files were deposited to SRA database^23^. For additional quality checks, the reads were next aligned to the TCH1516 genome (NC_010079.1) with Bowtie2. For each sample, at least 90% of reads successfully aligned to the genome^25^. Due to large number of collected samples, they were processed in 3 different batches. Each batch contained a full set of samples (i.e. all media types and all antibiotic concentrations) which allowed us to correct for any batch effect in the dataset. As a demonstration, we used the ComBat module within SVA package of R to correct for batch effect which resulted in highly reproducible data (Spearman r > 0.98) with close clustering of all biological replicates (Fig. 3b). The ComBat batch corrected TPM were deposited into GEO database^24^. As with ComBat, most modern differential expression analysis packages such as DESeq2 and EdgeR have built in ways to model these covariates.

### Bacterial Cytological Profiling

Prior to processing with CellProfiler, images must be manually screened for issues that would cause errors during image segmentation. Image adjustments such as contrast and brightness as well as background removal are used to reduce segmentation errors.

Following CellProfiler analysis, images of each object segmentation (13 in total) are generated and visually screened to ensure that measurements are obtained from the correct object. Additionally, prior to analysis, parameters from cell outlines must be combined with their related objects such as DNA. For each, a reference number is generated at the time of measurement and must match in both the cell and DNA objects ensuring that no cell-DNA mismatches are possible. A second checkpoint exists in the form of the “parent” tag for DNA objects in which the number of unique tags must match the number of cell outlines for that corresponding image. Excel files containing cellular features were uploaded to MassIVE^20^.

### Untargeted Liquid Chromatography Mass Spectrometry Data Acquisition

**Fig. 4:**
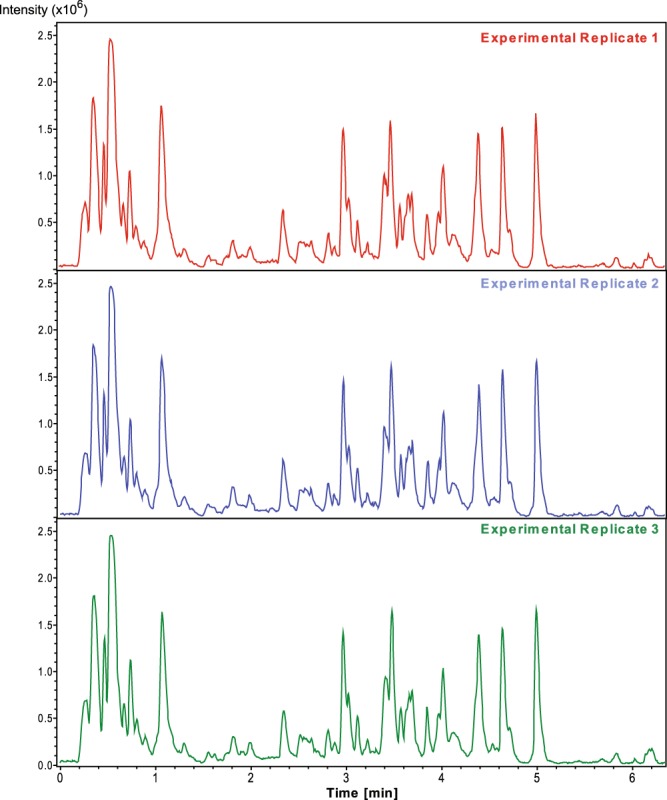
Validation of LC/MS base peak chromatograms of experimental replicates. (**a**) Evaluation of the reproducibility of both retention time and peak areas within the LC/MS data could be performed by direct comparison of the base peak chromatograms of experimental replicates. The plot represents a comparison of three separate experimental replicates of the same time-point from the same type of sample, in this case untreated TCH1516 grown in RPMI + 10% LB at 1 hr.

Global retention time reproducibility and ion intensity reproducibility was evaluated across all samples by direct comparison of both the base peak chromatogram (BPCs) and multiple extracted ion chromatograms (EICs) from each sample. Comparison of BPCs between experimental triplicate of each sample measured give an overall representation of the reproducibility of both the retention time and the peak intensity (Fig. 4). Additionally, the EICs for the various molecules determined to be present in each media could be evaluated across replicates to determine the more precise retention time drift (<0.1 minutes) and peak area (coefficient of variation <15%).

## ISA-Tab metadata file

Download metadata file

## Data Availability

The pipeline used to analyze RNA sequence data is available on figshare^21^. The script used to remove batch effects from RNA sequencing data is available on figshare^22^.
